# Changes in diet, activity, weight, and wellbeing of parents during COVID-19 lockdown

**DOI:** 10.1371/journal.pone.0248008

**Published:** 2021-03-03

**Authors:** Rachel G. Curtis, Timothy Olds, Ty Ferguson, François Fraysse, Dorothea Dumuid, Adrian Esterman, Gilly A. Hendrie, Wendy J. Brown, Rajini Lagiseti, Carol A. Maher

**Affiliations:** 1 Alliance for Research in Exercise, Nutrition and Activity, UniSA Allied Health and Human Performance, University of South Australia, Adelaide, SA, Australia; 2 UniSA Clinical and Health Sciences, University of South Australia, Adelaide, SA, Australia; 3 Health and Biosecurity, Commonwealth Scientific and Industrial Research Organisation, Adelaide, SA, Australia; 4 School of Human Movement and Nutrition Sciences, University of Queensland, Brisbane, QLD, Australia; University of Ottawa Heart Institute, CANADA

## Abstract

The COVID-19 pandemic has dramatically impacted lifestyle behaviour as public health initiatives aim to “flatten the curve”. This study examined changes in activity patterns (physical activity, sedentary time, sleep), recreational physical activities, diet, weight and wellbeing from before to during COVID-19 restrictions in Adelaide, Australia. This study used data from a prospective cohort of Australian adults (parents of primary school-aged children; n = 61, 66% female, aged 41±6 years). Participants wore a Fitbit Charge 3 activity monitor and weighed themselves daily using Wi-Fi scales. Activity and weight data were extracted for 14 days before (February 2020) and 14 days during (April 2020) COVID-19 restrictions. Participants reported their recreational physical activity, diet and wellbeing during these periods. Linear mixed effects models were used to examine change over time. Participants slept 27 minutes longer (95% CI 9–51), got up 38 minutes later (95% CI 25–50), and did 50 fewer minutes (95% CI -69–-29) of light physical activity during COVID-19 restrictions. Additionally, participants engaged in more cycling but less swimming, team sports and boating or sailing. Participants consumed a lower percentage of energy from protein (-0.8, 95% CI -1.5–-0.1) and a greater percentage of energy from alcohol (0.9, 95% CI 0.2–1.7). There were no changes in weight or wellbeing. Overall, the effects of COVID-19 restrictions on lifestyle were small; however, their impact on health and wellbeing may accumulate over time. Further research examining the effects of ongoing social distancing restrictions are needed as the pandemic continues.

## Introduction

The COVID-19 pandemic has dramatically and rapidly changed the lifestyles of people around the world. Restricted freedom of movement, limited access to health services, food providers and exercise resources, changes in social dynamics due to distancing, and psychological adaptations to isolation have all changed what we eat, our mental wellbeing, and above all the way we use our time. Indeed, a recent New Zealand study [1] surveyed 2002 people about their experiences during lockdown. Among participants’ open-ended responses, the most commonly used word was “time”.

Several surveys have been conducted which have straddled the period of lockdown, trying to capture changes in behaviour. Most have found reductions in levels of *physical activity*. A large international analysis examined step counts for >19 million days recorded by users of a physical activity app [2], finding that steps decreased by 6% after 10 days and 27% after 30 days of restriction. Declines after 30 days varied from 7% (Sweden) to 49% (Italy), apparently associated with the severity of lockdown. Other international studies have reported similar results; an analysis of Fitbit data from 30 million adults showed declines of 7–38%, most marked in the stricter lockdown zones of the European Union [3]. Similarly, a report from wearable manufacturer Withings of 2 million users of their products found that step count decreased by between 1% (Germany) and 56% (Hubei Province, China) [4]. An Australian consultancy found that 44% of adults had reduced their levels of physical activity, compared with 23% who reported increased physical activity. The declines tended to be greater among younger adults [5]. A UK survey of 2000 adults found that step count declined by 28% [6]. The declines also affected children; a study of 41 overweight children under strict lockdown in Verona found a decline in physical activity of 2 hours per week [7], while a study of 2427 children and adolescents in China showed a reduction in moderate-to-vigorous intensity physical activity of 435 minutes per week between January and March [8].

*Sleep* patterns have also changed, characterised by longer duration, later rising, more dreams and changes in sleep quality. The Withings study [4] found that device-measured sleep duration increased by 8–21 min per day across 8 countries. Adults woke up –1 to +32 minutes later, and experienced far fewer heart rate anomalies—suggestive of sounder sleep. The overweight children from Verona also slept an extra 30 minutes each day [7]. In a survey of 723 UK adults, 28% reported sleeping about the same as before the COVID-19 virus crisis, 49% reported sleeping more and 23% reported sleeping less [9]. Other studies have reported more frequent and more disquieting dreams [10]. A survey by Sleep Standards of 1015 US adults [11] found that respondents—especially younger adults—generally reported poorer quality sleep. According to retrospective self-report on the Insomnia Severity Index, the incidence of clinical insomnia among Chinese adults rose from 15% in October to December 2019 (pre-COVID) to 20% in February 2020 (during COVID) [12].

Most reports of changes in *screen time* have focused on children, and report very large increases. For example, in a US Parents Together survey, parents reported a doubling of online time from 3 hours per day to 6 hours per day. While this was partly due to online homeschooling, the main increases were on non-educational platforms such as TikTok and Netflix [13]. Italian children increased screen time even more—by 5 hours per day [7]. In a study of 254 Canadian families, parents reported that 87% children, 74% of mothers and 61% of fathers increased screen time since COVID-19 physical distancing restrictions were implemented [14]. Research in US adults has also shown increases in screen time of 30–40% [15].

There have also been reported changes in *diet*. In an April 2020 survey of 1005 American adults by the food and beverage communications firm Hunter [16], 54% said they were cooking more now than before the pandemic (vs 11% who reported cooking less), and 38% were ordering less takeout and delivery (vs 30% who reported ordering more). Among Canadian families, many parents reported spending more time cooking (mothers, 70%; fathers, 68%), but also reported eating more food (mothers, 57%; fathers, 46%; children, 42%) and eating more snack foods (mothers, 67%; fathers, 59%; children, 55%) [14]. On average, French adults consumed 235 kcal per day more during the first month of lockdown as compared to the month before lockdown [17]. Similarly, Italian children consumed one extra meal per day, along with more sugar-sweetened beverages and red meat [7]. While one Australian study of 270 alcohol drinkers [18] found no appreciable change in consumption, another nationally representative survey found that while people reported decreasing their alcohol consumption during lockdown, it was actually higher than in matched periods in previous years [19].

With reduced physical activity, higher screen time, and potentially poorer diet, it is unsurprising that some studies have reported increases in *weight*. A UK survey of 2000 adults found that the average self-reported weight gain from pre- to during lock-down was >2.5 kg [6]. Other studies have shown more modest change. For example, a survey of 339 Chinese adults found that average self-reported weight gain from 5 weeks before to during lockdown was 2.2 kg for females with BMI < 24, 1.7 kg for males with BMI <24, and 0.9 kg for females with BMI ≥ 24; however, males with BMI ≥ 24 showed average weight loss of 0.9 kg [20]. Additionally, among 4379 Spanish citizens surveyed between 1 and 4 weeks into lockdown, most (53%) reported no weight change, while 26% and 22% reported weight gain and weight loss, respectively [21]. Data from Withings automated scales found that adults around the world gained between 0.08 and 0.25 kg over about a month of lockdown [4].

*Mental health* also appears to have suffered under lockdown. A large Australian study of 15,000 respondents [22] found that reports of depressive symptoms had doubled or trebled, most markedly in younger adults. There were similar patterns for symptoms of anxiety [22]. In the UK, a study of 600 adults also found abnormally high levels of depression and anxiety, which were more pronounced amongst those who were more isolated [23]. Patterns were similar in New Zealand [24], Italy [25], Spain [26], the USA [27] and China [28]. Unsurprisingly, those with pre-existing conditions found that their mental health deteriorated under lockdown. A UK study by the charity Rethink Mental Illness found that 80% of people living with serious mental illnesses such as borderline personality disorder and psychosis thought that their mental health had become worse due to the impact of COVID-19 [29].

The ensemble of these studies reports decreases in physical activity, increases in screen time and sleep, later bedtimes, increases in weight, and poorer mental health. However, most of these reports come from the grey literature, based on retrospective self-report, or else derived from big data analytics with little individualised demographic information. The aim of the present study was to examine changes in 24-h activity patterns (physical activity, sedentary time, sleep), recreational physical activities, diet, weight and wellbeing in a group of adults who were intensively tracked both before and during the social distancing and lockdown periods of the COVID-19 response in Adelaide, Australia.

## Methods

### Study design

This study used data from the prospective cohort study “Annual Rhythms In Adults’ lifestyle and health” (ARIA). ARIA is an ongoing study that is following participants over a 13-month period, measuring daily activity, dietary intake, weight and wellbeing. The study was approved by the University of South Australia Human Research Ethics committee (Protocol number: 201901) and participants provided informed, written consent prior to commencing participation. The study has been registered on the Australian New Zealand Clinical Trial Registry (Trial ID: ACTRN12619001430123).

### Participants and procedure

A community-based sample of 64 healthy adults was recruited from greater metropolitan Adelaide, South Australia between September and November 2019. Parents and guardians of children enrolled in a separate three-year cohort study “Life on Holidays” [30] were invited to participate via email or postal invitation. Inclusion criteria were 18 to 65 years old, residing in greater metropolitan Adelaide, having access to a Bluetooth-enabled mobile device or computer and home internet, proficiency in English, and ambulant. Exclusion criteria were pregnancy, having an implanted electronic medical device, or experiencing or receiving treatment for any life-threatening condition impacting daily lifestyle and health. A baseline face-to-face home visit was conducted between October and November 2019 where participants were provided with a Fitbit Charge 3 activity monitor and Aria 2 body weight scale (Fitbit Inc, San Francisco, CA, USA), had their height measured, and completed a baseline survey about their demographics, health and lifestyle. Participants were asked to wear the activity monitor and weigh themselves daily, and to complete eight online surveys on dietary intake, work status, recreational activities, and wellbeing between December 2019 and December 2020.

This study used data from 61 participants who remained enrolled in April 2020. Survey data were timepoint 3 of the larger study, assessing behaviour and experiences in February 2020 prior to COVID-19 related restrictions in Australia, and timepoint 4, assessing behaviour and experiences in April when the most rigorous COVID-19 related restrictions were in place. Fitbit data were extracted for 14 days before (February 10–23 2020) and 14 days during (April 14–27 2020) the COVID-19 restrictions. In the latter period, restrictions included enforced social distancing, closure of cinemas and food venues, a ban on large gatherings, and widespread working from home and school closures, but did not involve shelter-in-place orders.

### Variables

#### Demographics

At baseline, participants reported their date of birth, sex, country of birth, marital status (never married, widowed, divorced, separated but not divorced, married or de facto), number of children at home, highest education level (below year 10, year 10, year 11, year 12 or equivalent, certificate III/IV, advanced diploma/diploma, bachelor degree, postgraduate or higher degree), combined gross household income (AU$; <$50,000, $50,000-$99,999, $100,000-$199,999, ≥$200,000), occupation (open-ended response classified according to the Australian and New Zealand Standard Classification of Occupations [31]), hours worked per week (none, <15, 15–35, 36+) and smoking status (yes, no). Height was measured at the baseline home visit (Leister Height Measure MKII).

#### Daily activity composition

Activity composition was measured using Fitbit Charge 3 activity monitors (Fitbit Inc, San Francisco, CA, USA) worn on participants’ non-dominant wrist 24 hours a day, except during water activities and device charging. Fitbit activity monitors are commonly used in biomedical research [32, 33] and have acceptable validity for moderate-to-vigorous physical activity (MVPA) [34], sleep [35] and total daily energy expenditure [36]. Each minute in every 24-h period was classified as sleep, sedentary, light, moderate or vigorous physical activity according to Fitbit’s proprietary algorithms, which also provided bedtime and get up time. Data were synced to participants’ Fitbit account and collected remotely via bespoke software called “Fitnesslink”, developed for the ARIA study. Minutes classified as sedentary (recorded when no other activities are detected) AND with missing heart rate data were classified as non-wear. Valid days were defined as a minimum of 18 hours wear time and overnight wear. Only records that contained at least 1 valid weekday and 1 valid weekend day during each 14-day period were used for analysis. Daily MVPA was calculated as the sum of minutes spent in moderate and vigorous activity. Daily activities (sleep, sedentary, light and MVPA) were calculated as the mean over each 14-day period, weighted 5:2 for weekdays and weekend days. Fifty-five participants were included in the analysis; on average they had 12 valid days in each period (Mdn = 12, IQR = 10–14 both timepoints) and 23 hours of wear time per day (before COVID-19 Mdn = 23.2, IQR = 22.8–23.5; during COVID-19 Mdn = 23.4, IQR = 22.9–23.6).

#### Recreational physical activity

Recreational physical activity was assessed using items from the How Areas in Brisbane Influence Health and Activity (HABITAT) study [37] which were modified to reflect recreational activity over the previous month. Participants rated how often they did 15 activities (e.g. running or jogging, team sports, water activities) on a 5-point scale (1 = never, 2 = once a month, 3 = once every 2 weeks, 4 = once a week, 5 = more than once a week).

#### Dietary intake

Dietary intake (energy including fibre, percentage of energy from protein, total fat, saturated fat, carbohydrate, alcohol and fibre) was assessed using the online Dietary Questionnaire for Epidemiological Studies (DQES v3.2; Cancer Council Victoria), which was modified to reflect diet over the previous month. The DQES v3.2 is a self-administered questionnaire that estimates nutritional intake based on 144 foods and beverages across five groups, including: cereal foods; sweets and snacks; dairy products, meats and fish; fruit; vegetables; and alcoholic beverages [38]. The DQES has been demonstrated to have good reproducibility and has good agreement with weighed food records [39].

#### Body weight

Body weight was measured using Fitbit Aria 2 smart scales (Fitbit Inc, San Francisco, CA, USA) [40, 41]. Participants were advised to weigh themselves daily in the morning, whilst wearing minimal clothing, prior to meals and after voiding. Body weight data was collected remotely via our Fitnesslink software. Weight was calculated as the mean of all measures taken over each 14-day period.

#### Wellbeing

Quality of life was assessed using the World Health Organization Quality of Life assessment 26-item version (WHOQOL-BREF) [42]. WHOQOL-BREF is a self-report questionnaire which measures four broad domains: physical health, psychological health, social relationships and environment. The WHOQOL-BREF has good discriminant validity, content validity and test-retest reliability and internal consistency [42, 43]. Internal consistency in the current sample pre-COVID was high (physical health r = .84; psychological health r = .82; social relationships r = .72; environment r = .76). Symptoms of depression, anxiety and stress were assessed using the 21-item short-form Depression Anxiety Stress Scale (DASS-21) [44]. The DASS-21 has good convergent and discriminant validity, adequate construct validity, and high reliability [44–46]. Internal consistency in the current sample pre-COVID was excellent (depression r = .94; anxiety r = .80; stress r = .92).

### Statistical analysis

Analyses used random intercept mixed effects models with family and individual IDs as the random effects to account for the structure of the data (repeated measures nested within individuals, nested within 45 families). Change over time in daily activity composition (expressed as isometric logratios) was examined using a multivariate linear mixed effects model, following recommendations by Baldwin et al [47] using the packages compositions [48] and lme4 [49] in R version 3.5.0 (R Foundation for Statistical Computing, Vienna, Austria). Changes over time in sleep timing, recreational physical activities, dietary intake, weight and wellbeing were analysed using linear mixed effects models using Stata 16 (StataCorp, College Station, TX, USA).

## Results

Participants were predominantly female, overweight, born in Australia, married and well-educated (Table 1). The sample was broadly representative of middle-aged Australian adults in terms of BMI (66% overweight or obese vs 66% of 35- to 44-year-old Australian adults [50]), smoking status (16% smokers vs 15% of Australian adults aged 18 and over [50]), whether they were born in Australia (72% vs 67% of 30- to 49-year-old Australian adults [51]), and marital status (79% married or defacto vs 74% of 34- to 44-year-old Australian adults [51]). However, the sample was overrepresented by women, parents (100% were parents whereas 77% of Australian women aged 30 to 49 years report having children [51]), and employed persons (84% reported working vs 75% of 35- to 45-year-old Australian adults [51]). The sample was also slightly more highly educated; although the sample had similar rates of non-school education (79% with a non-school qualification vs approximately 75% of 30- to 50-year-old Australian adults [52]), rates of University education were slightly higher (54% with a university degree vs approximately 40% of 30- to 50-year-old Australian adults [52]).

**Table 1:** Sociodemographic characteristics of the participants at baseline (n = 61).

Variable	N	(%)	Mean	(SD)
Age (years)			41.3	(5.8)
Female	40	(65.6)		
Male	21	(34.4)		
Weight (kg)			80.8	(18.6)
Height (cm)			169.6	(9.3)
*BMI*				
Underweight	1	(1.6)		
Normal	20	(32.8)		
Overweight	21	(34.4)		
Obese	19	(31.1)		
Current Smoker	10	(16.4)		
Born in Australia	44	(72.1)		
*Marital Status*				
Never married	7	(11.5)		
Married/defacto	48	(78.7)		
Separated, divorced or widowed	6	(9.8)		
*Number of children at home*				
One	4	(6.6)		
Two	29	(47.5)		
Three	21	(34.4)		
Four or more	7	(11.5)		
*Education*				
Year 10 or less	4	(6.6)		
Year 11–12	9	(14.8)		
Certificate/diploma	15	(24.6)		
University degree	33	(54.1)		
*Household income (AU$)*				
<$50,000	9	(14.8)		
$50,000 - $99,999	21	(34.4)		
$100,000-$199,999	22	(36.1)		
≥$200,000	9	(14.8)		
*Occupation*				
Managerial and professional	19	(31.1)		
Technical and clerical	13	(21.3)		
Community, personal service and sales	15	(24.6)		
Machinery operators, drivers, and labourers	3	(4.9)		
No job/other	11	(18.0)		
*Hours worked per week*				
None	10	(16.4)		
<15	3	(4.9)		
15–35	25	(41.0)		
36+	23	(37.7)		

Table 2 shows changes over time in daily activity composition, sleep timing, recreational physical activity, dietary intake, weight and wellbeing. There was a significant change in daily activity composition (χ2(3) = 8.931, *p =* 0.030). On average participants slept longer, got up later, and did less light physical activity during COVID-19 restrictions than before COVID-19 restrictions. Additionally, participants engaged in more cycling but less swimming, team sports and boating or sailing. Total energy intake did not change but participants consumed a slightly lower percentage of energy from protein and a greater percentage of energy from alcohol during COVID-19 restrictions. There were no changes in weight or wellbeing.

**Table 2:** Changes over time in daily activity composition, sleep timing, recreational physical activity, dietary intake, weight and wellbeing during COVID-19 restrictions (n = 61).

Variable	Before COVID-19		During COVID-19		Change	[95% CI]	Sig.^*a*^
*Daily activity composition*, *min(%)*^*b*^							0.03
** Sleep**	**497**	**(35)**	**523**	**(36)**	**27**	**[9, 51]**	
Sedentary	601	(43)	623	(43)	22	[-4, 40]	
** Light PA**	**318**	**(22)**	**269**	**(19)**	**-50**	**[-69, -29]**	
MVPA	25	(2)	25	(2)	1	[-7, 8]	
*Sleep timing*, *M(SD)*							
Bedtime	22:52	(0:59)	22:48	(1:18)	-2	[-24, 19]	0.835
** Get up time**	**07:04**	**(0:42)**	**07:40**	**(1:09)**	**38**	**[25, 50]**	**<0.001**
*Recreational PA*, *M(SD)*^*c*^							
PA with others in a park	2.7	(1.4)	2.4	(1.6)	-0.32	[-0.71, 0.07]	0.105
Running	2.2	(1.5)	1.9	(1.4)	-0.28	[-0.58, 0.02]	0.064
Weights	1.8	(1.5)	1.5	(1.1)	-0.31	[-0.66, 0.04]	0.083
** Cycling**	**1.6**	**(1.0)**	**1.9**	**(1.4)**	**0.35**	**[0.01, 0.69]**	**0.047**
Exercise class	1.5	(1.2)	1.4	(0.9)	-0.13	[-0.49, 0.24]	0.497
Golf	1.0	(0.3)	1.0	(0.1)	-0.03	[-0.10, 0.03]	0.324
** Swimming**	**1.6**	**(1.0)**	**1.1**	**(0.3)**	**-0.64**	**[-0.81, -0.27]**	**<0.001**
Tennis	1.1	(0.5)	1.2	(0.6)	0.04	[-0.13, 0.21]	0.621
** Team sports**	**1.6**	**(1.1)**	**1.2**	**(0.7)**	**-0.36**	**[-0.68, -0.05]**	**0.025**
Yoga, pilates, tai chi or qigong	1.4	(1.0)	1.4	(1.0)	0.05	[-0.15, 0.26]	0.601
Home-based exercises	2.3	(1.6)	2.6	(1.7)	0.32	[-0.11, 0.75]	0.147
** Boating or sailing**	**1.1**	**(0.5)**	**1.0**	**(0.1)**	**-0.13**	**[-0.24, -0.03]**	**0.015**
Water activities	1.0	(0.3)	1.0	(0.3)	-0.03	[-0.12, 0.06]	0.479
PA with others on a beach	1.5	(0.8)	1.4	(0.9)	-0.11	[-0.39, 0.18]	0.468
*Dietary intake*, *M(SD)*							
Energy including fibre (kj)	7048	(2107)	7091	(2080)	59	[-417, 536]	0.807
** Energy from protein (%)**	**19.5**	**(4.3)**	**18.8**	**(3.5)**	**-0.8**	**[-1.5, -0.1]**	**0.036**
Energy from total fat (%)	36.7	(5.3)	35.7	(4.7)	-1.0	[-2.1, 0.1]	0.085
Energy from saturated fat (%)	12.7	(2.8)	12.7	(2.7)	0.1	[-0.4, 0.6]	0.729
Energy from carbohydrate (%)	41.0	(6.4)	42.0	(5.6)	0.9	[-0.6, 2.5]	0.217
** Energy from alcohol (%)**	**2.8**	**(5.6)**	**3.7**	**(5.9)**	**0.9**	**[0.2, 1.7]**	**0.018**
Energy from fibre (%)	2.5	(0.8)	2.5	(0.7)	-0.1	[-0.2, 0.1]	0.248
Weight (kg), M(SD)	80.8	(18.6)	80.7	(18.6)	-0.0	[-0.4, 0.3]	0.906
*Wellbeing*, *M(SD)*							
WHOQOL physical health	16.2	(2.1)	15.9	(2.1)	-0.29	[-0.77, 0.18]	0.230
WHOQOL psychological	14.8	(2.3)	14.6	(2.5)	-0.17	[-0.61, 0.27]	0.453
WHOQOL social relationships	15.2	(2.9)	15.1	(2.9)	0.03	[-0.43, 0.48]	0.910
WHOQOL environment	15.8	(1.7)	15.6	(2.1)	-0.15	[-0.52, 0.22]	0.425
DASS depression	6.1	(7.7)	6.2	(8.0)	0.00	[-1.23, 1.23]	1.000
DASS anxiety	4.2	(5.7)	3.9	(5.8)	-0.26	[-1.19, 0.67]	0.584
DASS stress	9.3	(8.2)	10.0	(8.5)	0.53	[-1.02, 2.09]	0.510

^*a*^Based on linear mixed effects models adjusting for clustering of individual within family. The p value for the activity composition relates to the multiple correlation coefficient (Wald Chi-squared) for set of isometric log ratios (i.e., the change in overall composition).

^*b*^Time-use composition is presented as geometric means, adjusted to a sum of 1440 minutes and 100%. Bootstrapped 95% confidence intervals are presented for predicted change in time-use components.

^*c*^Recreational PA calculated based on 5-point ordinal scale where 1 = never, 2 = once a month, 3 = once every 2 weeks, 4 = once a week, 5 = more than once a week.

*Note*: Significant results are shown in boldface. PA = physical activity MVPA = moderate-to-vigorous physical activity, DASS = 21-item short form Depression, anxiety, stress scale; WHOQOL = World Health Organization Quality of Life assessment 26-item version.

## Discussion

The aim of the study was to comprehensively examine changes in daily activity patterns, diet, weight and wellbeing in a natural setting during an early period of COVID-19 restrictions in Adelaide, Australia. Small changes were identified in some aspects of diet (increased alcohol consumption and reduced energy from protein), and daily activity patterns (increased sleep duration, later get up time, and reduced light physical activity). Whilst participants reported some changes in recreational activities (namely, an increase in cycling, and a reduction in swimming, sport and boating), there were no overall changes in device-measured MVPA, body weight or measures of psychological wellbeing.

Before considering the implications of these findings, the strengths and limitations of our study must be acknowledged. Firstly, to our knowledge, this is the first study to examine numerous aspects of lifestyle (physical activity, sedentary time, sleep and diet), weight and wellbeing, in concert, during the COVID-19 pandemic. Fortuitously, we were already collecting high fidelity lifestyle and wellbeing data in a cohort study which commenced in late 2019, prior to the COVID-19 pandemic. Our data were collected prospectively with repeated within-person assessments, and used high-quality measurement methods, including device-measured weight and 24-h movement activities, meaning that we have excellent comparison data from before the COVID-19 pandemic. This is an important advantage over much of the research on lifestyle changes across the COVID-19 period, which has tended to rely on retrospective comparisons of lifestyle and psychological factors and is therefore highly susceptible to selection and recall bias.

The key limitation to our study is the relatively small sample size. Given this, we focused our interpretation on overall patterns, bearing in mind clinical significance. We also acknowledge that the small sample increases the likelihood that our participants may not be representative of the population of adults in Adelaide. A further limitation is that participants were middle-aged adults from one Australian city, who were predominantly female, though otherwise broadly similar to the general middle-aged Australian population in terms of demographic characteristics and weight status. One exception was that the participants were all parents of school age children, when about one quarter of Australian adults this age do not have children at home. It is unclear how our results might apply to adults without children. It is also unclear to what extent our results may be generalised to the other geographical areas, given differences in contextual factors within states of Australia and internationally (such as local COVID-19 social distancing restrictions, economic impacts and infection rates). For context, our COVID-19 data were collected approximately 1 month after the introduction of strict COVID-19 social distancing restrictions. At the time of data collection, restaurants, cafes, cinemas and playgrounds were closed, public and in-home gatherings were banned, some schools were closed (and where they were open, many families chose to keep their children home), and people were encouraged to work from home wherever possible. Whilst shops were allowed to remain open, many opted to close. The Australian government had announced financial assistance for people experiencing financial hardship related to COVID-19. Research with a larger and more diverse sample of adults in Adelaide is needed to confirm our findings, while broader research is needed to understand how conditions during lockdown affected adults in various Australian contexts.

In terms of people’s daily lifestyle activities, the largest changes were in participants’ sleep and sedentary patterns; around 30 minutes extra sleep and 20 minutes extra sedentary time each day, offset by a 50-minute reduction in daily light physical activity. The later get up time suggests that those who began working from home or had children learning from home during school closures may have replaced commuting time with sleep. Amongst adults, both long and short sleep durations are associated with detriments in health [53]. In our study, mean sleep duration both pre- and during COVID-19 was within healthy sleep guidelines (7–9 hours) [54] suggesting the changes we observed may not have either positive or negative health impacts. The reduction in light activity may reflect reduced active transport or a lack of incidental activity at work when people started working from home. On the other hand, the increase in cycling could be explained by attempts to avoid public transport among those who continued to travel to work. These changes in daily activities from pre- to during COVID-19 lockdown appear to mirror the differences in daily activities typically seen on weekday and weekend days; the latter are characterised by longer sleep durations, later wake-up times, and more sedentary time [55]. The changes we observed across the COVID-19 transition also resemble the differences between pre- and post-retirement, which in an Australian sample were characterised by 30 min longer sleep duration, and correspondingly later get up times, with no change in physical activity [56]. Although we saw few changes in participants’ recreational physical activities, we note that there was a floor effect for many activities. At both timepoints, over 80% of participants reported never doing exercise classes or yoga, and over 90% reported never doing golf, tennis, lawn bowls, boating or water activities. Therefore, little change would be expected in these activities.

Generally speaking, there were few changes in dietary intake. Overall energy intake did not change significantly, either statistically, or meaningfully based on absolute values (which suggested that energy intake was approximately 1% higher during lockdown than before). This contrasts with previous studies suggesting increased food intake in Canadian families [14] and increased energy intake in French adults during COVID-19 [17]. During lockdown, alcohol consumption increased. This is consistent with findings reported in another recent study [19]. However, even allowing for under-reporting, the increase amounted to less than half a glass of wine. With the increase in the percentage of energy from alcohol, there was a decrease in energy from protein, and there was a trend for percentage energy from fat to decrease (p = 0.07), though all these changes were fairly small.

Sleep aside, the changes in lifestyle patterns that we detected were generally in a direction associated with negative health impacts. It is possible that this suite of lifestyle changes, though small in magnitude, may have harmful impacts if sustained over a long period. This is particularly important when considered in the broader context of COVID-19. We are writing this study at a time that Australia, and many other countries around the world, is reinstating restrictions due to a second rise in infections. COVID-19 restrictions, in one form or another, are likely to remain for many months, and the impact of them on people’s health and wellbeing is likely to accumulate over time.

Somewhat surprisingly, there was no change in any of the measures of psychological wellbeing, either based on statistical results, or based on effect sizes. Effect sizes for change in WHOQOL-BREF scores from pre- to during lockdown ranged from Cohen’s d = -0.14 to 0.01. A previous study examining the WHOQOL-BREF’s responsiveness to change in 14 different patient populations reported that intervention effect sizes >0.2 were considered “small”, >0.5 “moderate” and >0.80 “large” [57], suggesting that the magnitude of changes seen in the current study were negligible. Similarly, effect sizes for change in the DASS-21 subscales ranged between d = -0.05 and 0.06, again suggesting negligible change. Ronk et al. (2013) [58] suggested that a change of approximately 4 points is considered meaningful change, whereas in our sample, the average change was less than a point. Average scores for depression, anxiety and stress were within the normal ranges at both pre- and during lockdown.

Our findings contrast with scientific discourse, with peer-reviewed commentaries predicting dire mental health sequelae [59, 60], such as a looming mental health “pandemic” [60], and with empirical studies showing high levels of distress during COVID-19 [25, 26, 28]. It also contrasts with empirical studies which have examined mental health in volunteer samples during COVID-19 and compared them with population norms [22–24, 27]. It is possible that selection bias may explain the difference in findings between those studies and ours–i.e. while people with heightened mental health symptoms volunteer for surveys on COVID-19 and mental health, our participants tended to have relatively good mental health (e.g., the majority of scores on the DASS-21 were in the normal range [44]). Our findings also differ markedly from two US studies that found that symptoms of depression and mental distress were significantly higher in nationally representative samples measured in April 2020 during the pandemic than those measured in 2018 [61, 62]. They also contrast with findings from a longitudinal probability sample of UK adults which showed a significant increase in mental distress between 2018–19 and April 2020 (1 month into UK lock-down) [63]. Our findings are perhaps even more surprising when considering that our sample was parents of primary-school aged children, who may have been contending with having children at home rather than at school. However, relative to many other countries, Australia had lower infection and mortality rates at the time of this study. Mental health would likely be more affected when the risk of catching the virus and becoming ill is greater, and when faced with critically ill family and friends. Additionally, lock-down was less strict in Adelaide as compared to the UK and many US states where non-essential stores were closed, and stay-at-home orders meant that people were likely to be more isolated from family, friends and colleagues. Furthermore, our study assessed only a short period of time. The effects of lock-down on well-being may become greater as lock-down extends. We also acknowledge that our sample was slightly more highly educated than population norms and may have experienced greater income security than other populations during this time. Participants were also middle-aged adults; a demographic that has reported enjoying the slower pace of life under COVID-19 restrictions [64].

In conclusion, this study comprehensively examined prospective changes in a small sample of middle-aged adults’ lifestyle behaviours and wellbeing across the early COVID-19 lockdown in Adelaide. It identified small dietary changes (increased alcohol consumption and reduced energy from protein), and modest changes in daily activity patterns (increased sleep duration and reduced light physical activity). No changes in MVPA, weight or psychological wellbeing were observed. Overall, results were more positive than might have been expected, however further rigorous research examining the health and lifestyle impacts of social distancing restrictions, particularly if they are ongoing, will be needed as the COVID-19 pandemic unfolds.
